# Regulation of Dendritic Cell Function in Inflammation

**DOI:** 10.1155/2015/743169

**Published:** 2015-07-02

**Authors:** André Said, Günther Weindl

**Affiliations:** Institut für Pharmazie (Pharmakologie und Toxikologie), Freie Universität Berlin, 14195 Berlin, Germany

## Abstract

Dendritic cells (DC) are professional antigen presenting cells and link the innate and adaptive immune system. During steady state immune surveillance in skin, DC act as sentinels against commensals and invading pathogens. Under pathological skin conditions, inflammatory cytokines, secreted by surrounding keratinocytes, dermal fibroblasts, and immune cells, influence the activation and maturation of different DC populations including Langerhans cells (LC) and dermal DC. In this review we address critical differences in human DC subtypes during inflammatory settings compared to steady state. We also highlight the functional characteristics of human DC subsets in inflammatory skin environments and skin diseases including psoriasis and atopic dermatitis. Understanding the complex immunoregulatory role of distinct DC subsets in inflamed human skin will be a key element in developing novel strategies in anti-inflammatory therapy.

## 1. Introduction

Dendritic cells (DC) are professional antigen presenting cells (APC), identified as key mediators between innate and adaptive immunities. Considering their heterogeneity, DC subsets display different phenotypic and functional properties, characterized by the distinct induction of tolerance or (auto)immunity [1–4]. While the recent years provided additional knowledge on the functional specialization of mouse subsets, our understanding of the immunoregulatory role of human DC populations is currently not well established. In particular, the specific function of human skin-resident DC including Langerhans cells (LC) and dermal DC (DDC) is still a matter of debate.

While functional skin DC research mainly focuses on mouse infection models or genetically modified mice, the availability of human DC populations is hampered by ethical and logistical aspects and the limited amount of pure and immature DC in excised skin explants. Despite these restrictions, human research is promoted, at least in part, by* ex vivo* differentiated subsets, which are utilized for the characterization of the predetermined antigen specificity of skin DC. However, a distinct functionality of LC and DDC in the presence of inflammatory cytokines has recently been suggested, underlying the heterogeneity of their contribution in skin-associated immunity. Additionally, in contrast to steady state, inflammation promotes the immigration of unique DC subsets and monocytes* in vivo*, thereby distinctively shaping immune responses under acute or chronic inflammation [5, 6].

This review aims to understand the functional characteristics of human DC populations in inflammatory skin environments, with focus on their distinct immunological properties in contrast to steady state conditions.

## 2. Dendritic Cell Subpopulations in Inflammatory Skin

The functional properties of mouse nonlymphoid tissue (NLT) resident DC subsets enhanced our current knowledge on DC-mediated immunity in skin, lung, liver, or intestine, especially under inflammatory conditions, whereas the organ-specific immune system in humans is still poorly understood. Research with human cells is mainly limited to* in vitro* generated DC derived from peripheral blood precursors and* ex vivo* characterization of primary subsets from excised tissues. To outline the present understanding of the DC network in human skin, we address critical differences in DC subtypes during inflammatory settings, compared to steady state, and further draw attention to the major differences in mouse skin immunity.

### 2.1. DC Network in Human Epidermis

LC are considered as the first immunological barrier, located in the epidermis, which perpetually arise from resident radioresistant precursor cells in steady state. It is evident that LC represent a long-lived subset as revealed by xenogeneic graft experiments and comprise about 1–3% of all nucleated cells in human epidermis [7, 8]. LC can be identified by a restricted pattern of surface molecules including CD1a, CD11c, CD32, FcɛR1, CD324, and HLA-DR and the specific expression of Birbeck granules and CD207 (Langerin). The latter are coexpressed by a distinct subtype of dermal resident DC in mice [9–11]. In humans, CD207^+^ DDC have been suggested [12]; however, only recently the presence of a small subset of myeloid DC in the dermis that express CD207^low^ has been confirmed [13].

The migratory potential of LC implicates their function as professional APC in steady state. Here, LC take up and process peptides and nonpeptides from their environment and subsequently present these fragments MHC-1 and MHC-2 dependent or via the CD1-receptor to local T lymphocytes or to T cells resident in skin draining lymph nodes, thereby contributing to the maintenance of tolerance towards self-antigens [14–16]. Considering their immunologic functionality, LC display a unique pattern of toll-like receptors (TLR) [17–20]. They appear susceptible towards viral pathogens, whereas the low or absent expression of TLR2, TLR4, and TLR5 possibly renders LC tolerogenic towards bacteria, thereby retaining the integrity of the nonpathogenic commensal skin flora [21–24].

While steady state epidermis exclusively harbours CD207^+^ LC, an infiltrating subset of DC is described for lesional skin of atopic dermatitis (AD), called inflammatory epidermal dendritic cells (IDEC), highlighting an altered DC composition during pathogenic settings [25]. IDEC are delineated from LC by the lower expression of CD1a and the lack of intracellular Birbeck granules and might originate from circulating blood monocytes. AD is a common, chronic, and highly pruritic skin disorder, characterized by increased numbers of infiltrating Th2 cells in acute lesions and a more predominant Th1 response in subacute to chronic forms [26]. Considering the interplay of LC and IDEC in human AD, both subsets contribute to a local inflammatory setting by the capture of allergens via IgE antibodies. This is promoted by the high expression of FcɛRI, together with low surface levels of Fc*γ*RII, thus revealing a phenotypic change of atopic DC subsets compared to healthy skin [27]. LC preferably contribute to the initiation of inflammation, at least for the extrinsic type of AD, by attracting eosinophils, monocytes, and T cells and promoting Th2 polarization. In contrast, IDEC presumably contribute to Th1 activity in more chronic lesions, by additionally releasing Th1-restricted cytokines [27, 28]. The prominent role of LC to induce Th2 activity in AD might be strictly related to the presence of thymic stromal lymphopoietin (TSLP) in skin lesions [29]. TSLP is secreted by surrounding keratinocytes (KC) due to an inflammatory skin environment, thereby distinctively shaping the immune responses of LC [30, 31]. The diverse impacts of locally secreted cytokines on the functional specialization of skin-resident DC will be discussed afterwards.

A prominent role for LC in the induction or maintenance of immunological events in psoriatic skin of humans is currently not determined [32]. Psoriasis is an immune-mediated inflammatory disease, causing typical lesions of skin and joints, called “psoriatic plaques.” The histological features comprise thickening of the epidermis and silvering scale, an increased vessel formation, causing an exaggerated vascularity, together with an infiltration of immune cells [33]. Increased numbers of activated CD45RO^+^ memory T cells and CD8^+^ cytotoxic T lymphocytes in psoriatic epidermis imply a mutual interplay with LC, that possibly regulates cytotoxic events and inflammatory cytokine release [34, 35]. Considering the human DC network in psoriasis, contrary to AD skin, overall numbers of LC in nonlesional psoriatic plaques remained unchanged [36, 37], although a decreased migratory capacity highlights their potential role in orchestrating local immune responses [36]. In contrast, a significant reduction of LC in lesional psoriatic epidermis and increased numbers in lesional dermis indicate an enhanced migratory capacity of LC during plaque formation [37]. The different migration pattern of LC in psoriasis might be explained by the existence of two LC types in humans.

LC composition under acute or chronic inflammatory conditions might be phenotypically and functionally more heterogeneous than originally assumed. Indeed, while LC are considered as a specialized subtype of DC, due to their unique origin and distribution, the repopulation of human LC under inflammatory conditions is likely distinct to steady state. Recent studies in mice revealed a transient immigration of blood derived Gr-1^hi^ monocytes into skin after induction of inflammation by UV-treatment that differentiate into short-term LC and repopulate the mouse epidermis in a first wave, being finally replaced by LC presumably derived from myeloid precursors [38]. These “short-term” LC revealed phenotypic differences compared to native LC, by lower expression of CD207, together with an altered distribution of class II MHC, suggesting an altered processing of viral antigens [39]. Although human studies are currently lacking, these findings outline the complexity of LC homeostasis during inflammation, hampering a discrete characterization of their functional properties.

It has to be taken into account that peripheral blood human monocyte-derived Langerhans-like cells (MoLC), which are conveniently used for functional studies* in vitro*, might not resemble steady state LC but have major similarities with LC derived from circulating monocytes during skin injury or inflammation [40]. Moreover, a very recent study revealed a potential precursor function for circulating CD14^−^ CD1c^+^ conventional DC (cDC), which possess the capacity to differentiate into LC-like cells* in vitro*, possibly providing a more physiological approach to generate human LC-like counterparts [41, 42]. However, whether human LC can really arise from peripheral blood monocytes or CD14^−^ CD1c^+^ blood cDC has not yet been demonstrated* in vivo* and a functional or transcriptomic comparison of MoLC, CD1c^+^ derived LC, and LC isolated from human skin is still missing.

### 2.2. DC Network in Human Dermis

Human dermis harbors at least three different subsets of DC, divided into CD1a^+^ CD11c^+^ CD14^−^  CD141^hi^ DDC, CD1a^+^ CD11c^+^ CD14^−^ CD1c^+^ DDC, and CD1c^+^ CD14^+^ DDC [43–46]. CD14^+^ DCs also express CD141, which is further upregulated during spontaneous migration from skin explant culture [47]. CD141^+^ DCs were initially identified in human blood [44, 48–50] and may act as immediate precursors of CD141^hi^ DC which can be found in low frequencies in the skin [43].

As stated above, resident CD207^+^ DDC have been identified in humans; however, in contrast to dermal CD207^+^ cells in mice, the human equivalent is related to CD1c^+^ myeloid DC but not CD141^hi^ XCR1^+^ (chemokine (C motif) receptor 1) DC [13].

The dermal CD141^hi^ subtype finds its counterpart in CD103^+^ and splenic CD8^+^ DC in mouse skin, whereas human CD1c^+^ CD14^−^ DDC resemble CD11b^+^ DC in skin and CD4^+^ DC in spleen of mice, respectively [51]. In contrast, the lineage belonging of the third subset, the CD14^+^ dermal DC, remains an obstacle. CD14^+^ DCs spontaneously migrate from excised human dermis and* in vitro* generated counterparts induce regulatory T cells through IL-10 release. However, they only poorly stimulate allogeneic T cell proliferation* in vitro* and their transcriptome and specific phenotype highlight their close relationship to human peripheral blood CD14^+^ monocytes and tissue resident CD14^+^ macrophages [52].

Their obvious monocytic origin might segregate the CD14^+^ DDC from CD14^−^ subtypes, which possibly arise from a DC-restricted bone marrow hematopoietic stem cell- (HSC-) derived precursor cell, which is currently identified as a pre-DC in mice [53, 54]. The existence of a common DC precursor (CDP) in mice separates the DC lineage from monocytes, whereas this is not yet confirmed for humans. Here, a recorded IRF8 gene mutation and four cases of DC, monocyte, and B and NK lymphoid- (DCML-) deficiency revealed a lack of circulating blood cDC and monocytes, together with reduced numbers of DDC [55, 56]. These findings highlight the presence of a common monocyte/DC hematopoietic precursor in human bone marrow and further suggest a strict dependency for the development of CD1c^+^ and CD141^+^ dermal DC* in situ* on circulating progenitor cells, namely, on their blood derived counterparts or even on monocytes [51].

Similar to LC, it is assumed that skin-resident DC take up and process self-antigens and contribute to the induction of tolerance during steady state. However, their immunologic function towards microbial pathogens, determined by the specific pattern of TLR, is particularly examined in human circulating blood DC. CD1c^+^ cDC express all TLR members, except TLR9, defining them as highly susceptible for a broad range of microbial antigens, whereas CD141^+^ subtypes lack TLR4, TLR5, TLR7, and TLR9 but highly express TLR3, thus rendering them as potent inducers of antiviral immunity [44, 57]. Considering their potential role as blood circulating precursor cells, these findings might also reflect the immunologic potential of skin-resident CD1c^+^ CD14^−^ and CD141^+^ DDC. Indeed, CD11c^+^ CD14^−^ HLA-DR^+^ skin DC, isolated from human dermis, express TLR1-8 and lack TLR9 and TLR10 [17].

Considering the complex network of different DDC subtypes, together with migrating LC in steady state dermis, the composition of immune cells under inflammatory conditions is even more complicated. The distinction between healthy skin and involved and uninvolved pathogenic inflammatory skin is crucial to understand the contribution of specific DC populations, especially in chronic skin diseases. It has to be considered that the most common skin diseases, AD and psoriasis, are characterized by an increase of DC numbers at the site of inflammation. It is challenging to dissect the infiltrating subsets from resident DC, due to the lack of specific markers, together with a well-described maturation of the involved DC, which is accompanied by an extensive genetic reprogramming and altered expression of phenotypic surface proteins, usually used for the characterization of distinct lineages [58].

Studies in mouse infection models uncovered the phenotypic plasticity of monocytes and DC, primarily present during inflammation, assuming a monocytic origin of inflammatory DC [59]. Similarly, the influx of monocytes into the site of inflammation might also contribute to an altered DC homeostasis in humans, but the functional relationship of human DC and monocytes is still unresolved [60]. In fact, an experimental proof is still missing demonstrating that DC can originate from monocytes* in vivo*, although the differentiation of peripheral blood monocytes into MoDC* in vitro* represents a common tool to characterize functional properties of human CD1a^+^ dermal DC [61, 62]. However, recent transcriptomic analyses revealed that the phenotypic and functional properties of MoDC are quite distinct to steady state DC, isolated from human tissues, but conversely highlight the possible relationship to inflammatory DC subsets, present in lesional skin of AD or psoriasis [63–65].

A hallmark of AD is the already mentioned infiltration of IDEC into the epidermis, whereas the majority of this population is found in the dermis. IDEC are thought to originate from infiltrating monocytes, thereby increasing the total amount of resident DDC and being responsible for the Th2 to Th1 switch in chronic lesions [25, 66]. Considering the composition of the DC network in psoriatic skin, the most prominent characteristic of either involved or uninvolved tissue is the presence of plasmacytoid DC (pDC) in psoriatic dermis [67]. pDC represent a population of circulating cells specialized in antiviral immune responses via TLR7 and TLR9 ligation and rapid release of type 1 IFNs [68]. pDC are normally absent in healthy skin and the infiltration of pDC into psoriatic dermis underlines the IFN-*α* signature in respective skin lesions. The topical administration of imiquimod, triggering TLR7-dependent induction of type 1 IFN release by pDC, leads to an exacerbation of psoriatic plaques [69]. A major contribution of pDC to the immunological events is mainly proposed in early psoriatic plaques, where high levels of chemoattractant molecules guide the infiltration of pDC into skin, whereas a less distinct role is assumed in chronic lesions [70, 71]. The depletion of pDC in DKO^*∗*^ mice (Jun^f/f^  JunB^f/f^  K5cre^ER^) prior to induction of psoriatic flares resulted in an attenuated plaque formation, whereas pDC depletion during active inflammation had no effect [37].

Dermal cDC subsets appear to be more involved in subacute to chronic phases of psoriasis. Chronically lesional plaques contain markedly increased numbers of CD11c^+^ DDC compared to uninvolved or healthy skin [72]. Although the number of the two common skin-resident CD11c^+^ subsets including CD1c^+^ DC and CD141^+^ DC remains unaltered, the immigration of CD11c^+^ CD1c^−^ CD141^−^ DC populations elevate the total number of DC in inflammatory skin [73]. Moreover, activated CD45RO^+^ memory T cells and CD4^+^ T lymphocytes are clearly increased in psoriatic dermis, resulting in a persistent interplay with resident DDC [35]. Among the infiltrating DC subsets, a population of dermal DC expresses 6-SulfoLacNac (slan), similar to a previously identified slan^+^ subset of circulating blood CD16^+^ monocytes, which might give also rise to slanDC in psoriasis [74, 75]. A second distinct subset of infiltrating DC is characterized by the potent release of TNF and iNOS, which is also a hallmark of an inflammatory DC population in mice, called TipDC, that play a major role for combating microbial challenge in skin infection models [76–78]. Mouse TipDC additionally display monocyte- and macrophage-related proteins, assuming their development from blood derived monocytes [79, 80]. Whether this can be extended for the human equivalent of TipDC is currently unknown. In fact, their pathogenic impact on psoriatic skin is thought to arise from the Th17 polarization capacity due to the enhanced IL-23 release and a local NO-mediated vasodilatation, together with the proinflammatory action of TNF on other skin-resident cells, including DDC and T cells [76, 81]. To what extent these environmental stimuli can orchestrate the immunological function of DC under inflammatory conditions is discussed in the following section.

## 3. Regulation of DC Function by Inflammatory Cytokines

The recognition of microbial pathogens is mediated by distinct pattern recognition receptors (PRR). As described earlier, TLR are differently expressed in skin-resident DC, rendering LC tolerogenic to the commensal skin flora, whereas especially CD1c^+^ DDC subsets express a broad pattern of TLR and are highly susceptible to a variety of invading pathogens [17–19, 44]. However, certain microbes are not specified by a single pathogen-associated molecular pattern (PAMP); thus the reactivity of DC is only insufficiently characterized by the individual TLR expression. DC are equipped with PRR distinct from TLR and the concomitant engagement partially contributes to synergistic or antagonistic effects, as a consequence of an interaction of receptor-dependent signaling pathways. A complex interplay between different PRR is already recognized that leads to changes in innate and adaptive immune responses. For instance, the C-type lectin receptor (CLR) CD209/DC-SIGN induces a critical cross-talk with TLR4 [82].* Mycobacterium tuberculosis*-derived lipoarabinomannans are recognized by CD209, inducing the phosphorylation of nuclear factor-*κ*B (NF-*κ*B) subunit p65, previously activated by the TLR4/MyD88-dependent pathway, thereby enhancing the DNA binding capacity and transcription rate of the IL-10 promoter. Interestingly, this anti-inflammatory mechanism is not described for TLR4 ligation alone; thus the diminished capacity of CD209^+^ DC to induce an adaptive immune response partially explains the cellular escape mechanisms of* M. tuberculosis* [83]. In contrast, concurrent TLR-dependent activation of CD1c^+^ DC, isolated from human blood PBMC, can amplify the immunologic response. Here, DC increase the IL-12 release when simultaneously stimulated with LPS and the TLR7/8 ligand R848, compared to TLR4 ligation alone [84].

Consequently, the common use of single molecular stimuli for selective PRR to characterize the activation of human DC or monocyte-derived counterparts, might not resemble their role* in vivo*. This becomes even more meaningful, when considering the surrounding cytokine and chemokine environment, which evidently affects the function of local skin-resident DC. Besides their relevance in the protection against invading microbes, DC are highly susceptible to damage-associated molecular patterns (DAMP), derived from surrounding KC, fibroblast, and other skin-resident immune cells. Here, self-nucleotides, heat shock proteins, antimicrobial peptides (AMP), and inflammatory mediators, namely, type I and II IFNs, TNF, and IL-1*β*, critically orchestrate the functionality of DC [85–87]. Notably, DC themselves are potent producers of proinflammatory cytokines and they use these mediators to communicate with other skin cells [88]. The regulation of the surrounding environment, presumably by a similar intracellular cross-talk previously described for different PRR, is obvious for TNF and most notably the IL-1 receptor family. The latter shares common similarities and significant homologies with TLR-dependent pathways, that leads to the activation of mutual adaptor proteins and transcription factors, including MyD88, TRAF-6, and NF-*κ*B [89].

The release of IL-1 cytokines or TNF is a major factor in the maturation process of DC and the regulation of immune responses by skin-resident DC is critically dependent on the type of stimulus together with the environmental setting. Therefore, compared to the PRR-dependent cross-talk, the role of skin-resident DC appears even more complex in the presence of an arsenal of pro- and anti-inflammatory mediators.

### 3.1. Regulation of Maturation/Activation

Considering the synergistic effect of LPS and R848 on the IL-12 release by human blood CD1c^+^ DC, it is worth mentioning that the addition of the proinflammatory cytokine IFN-*γ* further enhances the secretion of IL-12, whereas IL-4 leads to a decrease of cytokine production, confirming its inhibitory effect on Th1 polarization [84]. In accordance with the high expression of intracellular TLR3, CD141^+^ DC, isolated from human skin, display a strongly activated phenotype in response to poly(I:C), assessed by the expression of CD80 and CD86, and additionally secrete IL-6, CXCL-10, and TNF, while the combination with inflammatory mediators, including TNF, IL-1*β*, IFN-*α*, and IFN-*γ*, additionally induces the release of IL-12 [43, 44]. CD1c^+^ DC reveal a wide array of secreted cytokines in response to poly(I:C), namely, IL-6, IL-8, CCL-3, CCL-4, CCL-5, CXCL-10, TNF, and IL-1*β*, whereas the latter two may provide the necessary stimuli for CD141^+^ DDC to produce IL-12 [44, 57]. Similarly, a critical role for IL-1*β* in driving the IL-12 release by activated MoDC in a concentration-dependent manner is described, thus potently enhancing a proinflammatory immune response [90]. Consistent with this, human immature MoDC and MoLC reveal distinct maturation-related processes, including the expression of CD83 and CD86 and the release of Th1- and Th17-restricted cytokines upon stimulation with different TLR ligands in the presence or absence of TNF and IL-1*β* [24, 91].

The development of human LC strictly depends on transforming growth factor-*β* (TGF-*β*), a cytokine expressed by KC and LC [92–94]. Interestingly, besides its modulatory effects on LC homeostasis, KC growth, and differentiation, TGF-*β* is a pleiotropic cytokine with evident anti- and proinflammatory capacities, able to orchestrate the differentiation of regulatory T lymphocytes (Treg) and inflammatory Th17 cells in a concentration-dependent manner [95, 96]. In fact, human LC constantly receive modulatory signals in an autocrine and paracrine manner due to the high levels of TGF-*β* in the epithelium. However, under physiological conditions, TGF-*β* might render human LC tolerogenic towards a wide range of skin commensals and inhibits their final maturation and migration to regional lymph nodes [97, 98]. The immunosuppressive effect of TGF-*β* is primarily mediated by the inhibition of p38 mitogen-activated protein kinase (MAPK), which leads to hyporesponsiveness to various stimuli LC regularly receive in human epidermis, including haptens, ATP, and UVB [99]. However, in contrast to physiological conditions, enhanced TGF-*β* production by KC during inflammation might alter LC and DC maturation, trafficking, and initiation of immunity. In fact, an augmented contact hypersensitivity (CHS) response in TGF-*β*-overexpressing transgenic mice indicates a critical role for TGF-*β* in orchestrating skin immunity by cutaneous DC [100].

### 3.2. Regulation of Migratory Capacity

TGF-*β* critically controls the function of human LC under steady state conditions, whereas higher concentrations of this cytokine reveal its pleiotropic character, inducing enhanced migration of skin-resident DC. However, the migratory capacity is critically dependent on the local chemokine milieu and the expression of distinct chemokine receptors, namely, CCR7, mediating the chemotaxis of DC towards skin draining lymph nodes [101]. The unique location of LC in the epidermal layer of human skin indicates a distinct migration pattern compared to other DC subsets and is described as a two-step model. Chemotactic studies on human skin explants, epidermal sheets, and LC derived from the MUTZ-3 cell line (MUTZ-LC) reveal a strict dependency on CXCR4 expression, mediating the initial emigration of LC into dermis, whereas CCR7 governs lymph-node homing to a later time point [102–104].

The impact of TGF-*β* on the surface expression of chemokine receptors on immature MoDC* in vitro* comprises increased levels of CCR1, CCR3, CCR5, CCR6, and CXCR4 and decreased CCR7 mRNA expression, even in the presence of TNF [105]. This distinct receptor profile induces chemotaxis towards CCL5, CCL20, and CXCL12, critical chemokines mediating the maintenance of cutaneous DC in human epidermis and dermis, respectively [102, 106]. Accordingly, the lower migration towards CCR7-ligand CCL19 in TGF-*β* treated mature MoDC, compared to stimulation with TNF alone, assumes an opposing role for both cytokines in lymph-node homing of mature skin DC [105].

TNF is a critical inducer of DC migration in mouse skin and human skin organ cultures [107]. In addition, LC-derived IL-1*β* is assumed to be a pivotal stimulus to reduce DC numbers in mouse skin [108]. Moreover, TNF and IL-1*β* augment the spontaneous migration of DC, whereas the use of inhibitory monoclonal antibodies (mAB) abrogates this effect and reveals an interdependence of both cytokines on the migratory capacity [109]. Here, anti-TNF mAB inhibit the IL-1*β*-mediated chemotaxis and vice versa, indicating a critical cross-talk during the initiation of DC migration.

Although the above-mentioned TGF-*β*-mediated regulation of chemokine-receptor expression in MoDC is described in a dose-dependent manner, the* in vitro* assessment of chemotactic properties does not sufficiently consider the local cytokine and chemokine milieu* in vivo*. The steady state migration of human LC maintains immunological tolerance to self-antigens; thus a CCR7-mediated migration to skin draining lymph nodes remains possible despite the continuous presence of TGF-*β*. Moreover, in inflammatory skin conditions, the proposed functional antagonism of TNF and increased concentrations of TGF-*β* do not explain the augmented CHS responses in TGF-*β*-overexpressing transgenic mice, where an increased DC trafficking indicates an enhanced expression of CCR7 [100]. Thus, additionally surrounding skin-resident cells and the local cytokine and chemokine milieu, respectively, might contribute to the enhanced maturation and chemotaxis of cutaneous DC to regional lymph nodes.

### 3.3. Regulation of T Cell Response

In contrast to macrophages, the most potent phagocytes in human skin, DC are unique in their ability to link innate and adaptive immunity and are therefore not primarily involved in the local clearance of pathogens. The cellular mechanisms that regulate their maturation process, including uptake, processing, and presentation of foreign antigens to T cells, are more important attributes to define DC function.

LC isolated from human epidermis are powerful inducers of allogeneic T cell proliferation* in vitro* and preferentially differentiate naïve CD4^+^ T cells into lymphocytes, secreting Th2-related cytokines, including IL-4, IL-5, and IL-13 [110]. Compared to LC, CD14^+^ and CD1a^+^ DDC are less potent inducers of Th2 polarization. CD14^+^ DDC prime CD4^+^ T cells into follicular T cells (Tfh), inducing the switch of naïve B cells into plasma cells, thus elevating the levels of IgM, IgG, and IgA. Furthermore, CD1a^+^ DDC promote the proliferation of allogeneic CD8^+^ T lymphocytes more potently than CD14^+^ DC. However, LC possess the highest capacity to induce a polarization into cytotoxic effector T cells (CTL) and are thereby defined as effective inducers of humoral (Th2) and cellular (CTL) immunity. In fact, this distinctive potential to prime different effector T cells is a consequence of the specific cytokine expression profile [111]. While IL-15, secreted by LC, is localized at the immunologic synapse with CD8^+^ T cells and enhances the differentiation into CTL, the addition of IL-10, usually produced by CD14^+^ DDC, inhibits the respective polarization. This is even amplified in the presence of TGF-*β*, highlighting the modulatory capacity of the pleiotropic cytokine on human LC.

Interestingly, transcriptional profiling of human and mouse skin DC subsets revealed a functional overlap of human LC with mouse CD103^+^ CD8^+^ DC, rather than with mouse LC, suggesting fundamental discrepancies in LC function in humans and mice [112]. Human LC are strongly enriched in genes associated with cross-presentation, potently resulting in efficient cross-priming of T cell clones. LC induced higher IFN-*γ* secretion upon cross-presentation to allogeneic CD8^+^ T cells, compared to lymphocytes primed by other skin-resident DC. This highlights the clinical importance of human LC as specific target of intradermal vaccination with soluble tumor antigens, inducing protective CD8^+^ T cell responses.

The composition of human skin DC subsets is insufficiently accurate by the separation into CD14^+^ and CD1a^+^ DDC, since the latter consist of two distinct populations, namely, CD1c^+^ DC and CD141^+^ DC [43, 44, 57]. However, the characterization of the distinct subsets is largely limited to* in vitro* studies of circulating counterparts. Primary cells isolated from human blood induce proliferation and priming of naïve CD4^+^ T cell, although poly(I:C) stimulated CD141^+^ DC reveal a higher capacity than CD1c^+^ DC to induce Th1 responses [44, 57]. Additionally, both subsets induce proliferation and differentiation of naïve allogeneic CD8^+^ T cells upon stimulation with poly(I:C) [57]. Moreover, CD141^+^ DC and CD1c^+^ DC drive the production of IL-2, but little or no production of the Th2-related cytokines IL-4 and IL-5 or the regulatory cytokine IL-10 is indicated. Finally, CD1c^+^ DC isolated from human blood are more potent inducers of IL-17 production by autologous CD4^+^ T cells, indicating Th17 differentiation [113].

Proliferation and polarization of distinct T cell subsets are critically dependent on the type of stimulus, the uptake, processing, and presentation of antigens and the individual cytokine pattern, released by DC. Indeed, primary CD1c^+^ and CD141^+^ DC, isolated from human blood, similarly express type I MHC and present peptides to CD8^+^ T cells [44]. Considering their capacity to cross-present proteins to CD8^+^ T cells, unstimulated CD1c^+^ DC appear more capable of cross-presenting model antigens, compared to immature CD141^+^ DC, whereas the latter significantly enhance their cross-presenting capacity upon activation with poly(I:C) and are superior to CD1c^+^ DC. Regarding the selective expression of TLR3 and CLEC9A, a specialized activity of CD141^+^ DC is recognized in cross-presenting endogenous material from dying cells, whilst recent findings finally confirmed this assumption by demonstrating cross-presentation of pp65-protein, derived from necrotic cells [44]. Human blood CD1c^+^ and CD141^+^ DC further reveal a similar capacity to internalize fluorescent Lucifer yellow and to process and present human model antigens to autologous CD4^+^ T cells* ex vivo*.

In contrast, LC isolated from human skin display a reduced capacity to take up bacterial antigens due to the low expression of CD32 and further reveal a deficiency in processing and presenting restricted antigens on MHC-II receptors [23]. Consequently, LC appear incapable of efficiently restimulating memory CD4^+^ T cells but in contrast induce the polarization of Foxp3^+^ Treg. Again, these specialized properties, together with the particular TLR expression profile, define LC as valuable arbiters of the integrity of cutaneous commensals in steady state. However, human MoLC demonstrate different immunological functions and properties in the presence of inflammatory cytokines and TNF and IL-1*β* might override the unresponsiveness towards bacterial agonists [24]. Consistent with the lack of TLR4 expression, MoLC are unable to prime naïve allogeneic CD4^+^ T cells into INF-*γ* secreting lymphocytes in response to LPS, whereas the combination with TNF and IL-1*β* induces strong Th1 polarization. Although it is currently not confirmed for human LC derived from skin-resident precursors, this addresses the insufficient activation of intracellular pathways, rather than the individual TLR expression, proposing a cross-regulation of receptor-dependent signaling.

Alterations of DC-mediated T cell priming capacities are also described in screening experiments, testing different cocktails of TLR agonists and cytokines, such as LPS, poly(I:C), R848, INF-*γ*, IL-1*β*, and TNF [114]. Differently stimulated human MoDC reveal an inconsistent capacity to induce allogeneic CD4^+^ T cell proliferation, whereas differences in the promoted T cell response are evident by analysis of IL-13, IFN-*γ*, and TNF. While the cytokine mixture of IFN-*α*, IFN-*γ*, poly(I:C), and IL-1*β* most effectively induces IFN-*γ* release by CD4^+^ T cells, no TLR/cytokine combination promotes Th2 differentiation by mature MoDC.

The screening of different activation mixtures is important for the clinical implication of DC in prospective therapies. Manipulation of DC-mediated immunity is already under consideration for vaccination strategies, targeting skin DC by delivering antigens to specific receptors, including CD205, CD206, or CD209 [115–118]. Distinct supplements enhance the effectiveness of vaccination therapies, including the TLR agonist LPS and aluminum compounds, which are used most frequently as vaccination adjuvants [119, 120]. In fact, particles of aluminum salts are recognized via the NOD-like-receptor (NLR) family, especially their intracellular assemblage, the inflammasomes [121, 122]. This cytoplasmic complex senses intracellular danger signals and induces the cleavage of pro-IL-1 cytokines in its active forms via caspase 1 and the subsequent release of potent inflammatory mediators [123, 124]. Thus, DC-derived IL-1 cytokines, including IL-1*α*, IL-1*β*, IL-18, and IL-33, may act as major enhancers of vaccine-based immune responses.

### 3.4. Regulation of Inflammatory Skin Diseases

The role of DC in chronic inflammatory skin diseases is markedly controlled by the local environment. Skin-resident cDC, as well as infiltrating populations, are mediating the induction and maintenance of psoriasis and AD, thereby critically contributing to the individual cytokine pattern. In fact, both skin diseases are characterized by the biphasic presence of distinct T lymphocyte populations, namely, Th1/Th17 in psoriasis and Th2/Th1 in AD. Interestingly, despite the distinct Th profiles* in vivo*, isolated DC from skin lesions of psoriasis and AD patients display a similar T cell polarizing capacity* ex vivo* [125]. However, both subsets differ in the release of specific chemokines, able to recruit different memory T cell subsets into the skin. This underlines the importance of the local pathogenic cytokine and chemokine pattern to modulate the function of DC.

Given their capacity to initiate a cascade of immune responses, DC of both plasmacytoid and myeloid lineage are thought to display a pathogenic role in psoriasis [81]. The interplay between DC, T cells, and KC gives rise to a self-perpetuating loop, amplifying and sustaining inflammation. The local cytokine profile of psoriatic skin mediates an enhanced and persistent activation of DC, driving the activity of Th1 and cytotoxic Tc1 cells or Th17/Tc17 lymphocytes, by secretion of TNF, IL-12, and IL-23 [126, 127]. The most prominent characteristic of psoriatic skin is the presence of pDC as a consequence of chemerin expression in early plaques [70]. In patient biopsies, only low numbers of pDC are present in nonlesional skin but are significantly increased within lesional psoriatic skin [37].

Importantly, self-nucleotides, released from stressed and dying skin cells, are forming immunogenic aggregates with the cathelicidin LL37, an AMP produced by activated neutrophils and KC in response to pathogens. LL37/self-RNA complexes promote a TLR7- and TLR9-dependent stimulation of pDC, inducing a massive release of type 1 IFN via activation of the IFN regulating factor-7 (IRF-7) [67, 128, 129]. INF-*α* is a potential pathogenic cytokine in the initiation of psoriasis and is capable of stimulating local bystander DDC, which themselves produce IL-12 and drive the polarization of Th1/Tc1 lymphocytes [33]. Interestingly, studies in the imiquimod-induced psoriasis mouse model revealed a pivotal role of dermal cDC in the formation of psoriatic skin lesions [130]. The selective activation of TLR7 on CD11c^+^ DDC induced local skin inflammation independent of pDC and the presence of type 1 IFN, whereas Langerin^+^ DC, including LC, were dispensable during imiquimod-induced psoriasis. In contrast, the absence of LC but not other Langerin^+^ APC led to psoriasis aggravation in DKO^*∗*^ mice, possibly by production of immunosuppressive IL-10 specifically by LC [37]. Thus, although pDC are necessary for the initiation of psoriasis, the specific role of other skin-resident DC subsets in the attenuation or exacerbation of psoriasis largely remains elusive.

The psoriatic infiltrate is further composed of inflammatory DC expressing TNF and iNOS [67, 76]. The TNF receptor is expressed in a wide range of cells and upregulated in psoriasis [131]. Its engagement induces the expression of intracellular adhesion molecule-1 (ICAM-1), facilitating the diapedesis of circulating leukocytes, and further stimulates KC and dermal fibroblasts to produce the neutrophil chemoattractant IL-8, as well as IL-6 and IL-1*β* [132–134]. In addition, the immunogenic complex, consisting of LL37/self-RNA, directly induces cDC activation leading to the differentiation into mature DC and the production of TNF and IL-6 [128]. In fact, normal skin-resident cDC from psoriatic lesions are positioned immediately beneath the hyperplastic epidermis and surrounded by T cells and, compared to DDC from healthy skin, are more effective in spontaneous stimulation of autologous T lymphocytes into Th1 and cytotoxic Tc1 cells [135]. Moreover, it is well accepted that Th1-derived IFN-*γ*, in interaction with IFN-*α* and TNF, is a key cytokine in the pathogenesis of psoriasis and is further described to stimulate DDC to produce IL-1 cytokines and IL-23, both critical mediators of Th17 polarization, the major subset in chronic psoriatic plaques [33, 136–138]. Finally, slanDC, another inflammatory DC subset found in psoriasis, also induce the release of IL-1*β*, IL-6, TNF, IL-12, and IL-23 in response to LL37/self-RNA complexes [74].

The potent release of IL-23 by inflammatory DC and activated cDC, together with IL-6 and IL-1*β*, mainly derived from surrounding skin cells, promote the polarization of T lymphocytes in chronic psoriatic plaques. Here, Th17 cells orchestrate the typical cytokine pattern of skin lesions, consisting of CCL20, IL-6, IL-17A, IL-17F, IL-21, IL-22, and IL-26 [139–141]. Consistent with this, mouse data suggest that TLR7-mediated activation of Langerin^−^ cDC triggers psoriatic plaque formation* in vivo* via activation of inflammatory Th17 lymphocytes [130]. The importance of IL-23 as therapeutic target is further highlighted by observations that inhibition of IL-23R signaling ameliorated disease symptoms in DKO^*∗*^ mice [37]. It is noteworthy that IL-4 therapy improves psoriasis in humans [142] by suppressing IL-23/Th17 responses without blocking IL-12-dependent Th1 responses [143].

Moreover, IL-17A/IFN-*γ* or IL-17/IL-22 double-producing T cells have been described in the skin and blood of psoriasis patients [144–146]. Besides Th17 cells, other cellular producers of IL-17A are known, including CD8^+^ Tc17 cells, *γδ* T lymphocytes, and natural killer (NK) T cells [147–149]. IL-17A and IL-17F are potent recruiters and activators of neutrophils, thereby linking adaptive and innate immunity, whereas the IL-17A-induced release of CCL20 by KC maintains the presence of T cells in psoriatic skin [145, 150]. Thus, key processes during disease maintenance include the release of IL-12 and IL-23 by dermal cDC and inflammatory subsets, the production of proinflammatory mediators such as IL-17A, IL-17F, and IL-22 by activated Th17/Tc17 cells and IFN-*γ* and TNF by Th1/Tc1 cells, and the continuous release of chemotactic factors, driving the infiltration of immune cells into the skin [33, 81, 151]. Likewise, inflammatory mediators stimulate psoriatic KC to produce and release AMP, such as LL37, S100 proteins, and *β*-defensins, a variety of chemotactic factors, and inflammatory cytokines, including IL-1*β*, IL-1*α*, IL-6, IL-15, IL-18, and IL-20, thus providing a positive feedback mechanism and amplifying the ongoing immune response [152].

In contrast to psoriasis, the typical cytokine expression pattern of AD is characterized by a local increase of IL-4, IL-5, IL-13, TSLP, and IgE, driving DC to induce a biphasic T cell profile by the secretion of chemotactic factors, involved in Th2 recruitment and the release of Th2/Th1-polarizing cytokines in chronic lesions [126]. As a consequence of microbial challenge or pathologic skin conditions, a disrupted or altered skin barrier function induces a persistent local inflammation. AD-related skin abnormalities appear to be associated with mutations in the filaggrin gene, encoding a structural protein essential for skin barrier formation, and thus in contrast to normal skin allow the penetration of allergens and microbes into deeper skin layers [153]. In AD, LC express high levels of FcɛRI, recognize IgE-bound antigens, and subsequently release IL-8, CCL2, and IL-16* in vitro*, thereby recruiting eosinophils, monocytes, and T cells [66, 154, 155]. Furthermore, LC play an important role in allergen presentation to Th2 cells and promote hyperstimulation of Th2 immunity [156]. The increased expression of FcɛRI on LC is related to the enhanced expression of the FcɛRI*γ* chain, which is assumed to be preserved by increased IgE level in AD patients [157]. Moreover, compared to psoriatic skin, the levels of CCL5, CCL11, and monocyte-chemotactic protein-4 (MCP-4) are highly increased and thus additionally contribute to the immigration of CCR3 expressing eosinophils and macrophages into AD lesions [158]. The Th2 cytokine pattern with high levels of IL-4, IL-5, IL-13, and IL-31 further creates an amplification loop, where IgE production is induced and FcɛRI-bearing cells perpetuate the inflammatory cycle [66, 159, 160].

The prominent role of Th2 cells in the initial phase of AD is closely related to the high expression of TSLP in AD skin. This IL-7-related cytokine is strongly secreted by surrounding KC and induces the allergen-independent maturation by increasing the expression of CD83, CD86, and MHC II on both LC and CD11c^+^ DC [29]. The precise mechanisms, leading to the enhanced TSLP release, are currently under investigation, but microbial antigens, cellular stress signals, and proinflammatory cytokines might critically contribute to its production [30, 31, 161]. In airway epithelial cells, IL-1 cytokines or TNF synergized with Th2-related cytokines IL4, IL-5, and IL-13 to induce the expression of TSLP, indicating a strict dependency on the NF-*κ*B promoter [162]. Besides KC and epithelial cells, also neutrophils, IgE-stimulated mast cells, or macrophages are considered as potent inducers of TSLP secretion [29, 163]. However, the capacity of TSLP-activated LC to polarize T cells into proallergic Th2 lymphocytes, as described* in vivo*, is critically dependent on the local cytokine milieu.* In vitro* studies revealed that the expansion and differentiation of autologous CD4^+^ T cells into a Th2 subtype were skewed to a Th1 polarization by the presence of IL-12 [164, 165]. Nevertheless, TSLP further drives skin DC to produce IL-8, IL-15, CCL17, CCL22, and CCL24, chemotactic factors mainly attracting Th2 cells. Therefore, the expression of FcɛRI on LC, together with the local expression of TSLP and Th2-attracting chemokines, comprises pivotal components to favor a Th2 signature* in vivo* [166].

The inflammatory DC subset found in AD is quite distinct from psoriasis without iNOS signature or high TNF production [167]. According to the current hypothesis, IDEC presumably derive from blood circulating monocytes and are recruited into acute AD lesions by signals mediated from cells of the inflammatory micromilieu, due to their expression of skin-homing receptors CCR5 and CCR6 [168, 169]. However, IDEC also express FcɛRI and thus respond to specific IgE-bound antigens and drive allergen presentation to T cells. In contrast to LC,* in vitro* stimulated “IDEC-like” cells mainly produce IL-1, CCL20, IL-16, and Th1-related cytokines IL-12 and IL-18, thus promoting Th1 polarization and IFN-*γ* production [66]. Therefore, IDEC are primarily responsible for the maintenance of AD to chronic lesions, characterized by a more pronounced Th1 cytokine profile.

In addition to Th2/Th1 activity, Th22 cells appear in high numbers in lesional AD skin, where a correlation between disease severity and increase of Th22 cells is described [170, 171]. In acute and chronic lesions, Th22 cells are thought to be activated by LC, whereas IDEC primarily mediate a Th1 response [125, 172]. However, as already shown in psoriatic plaques, it is expected that other skin-resident cells, namely, *γδ* T cells, innate lymphoid cells, mast cells, macrophages, and even DDC, are also potent producers of IL-22, thereby possibly contributing to Th22-related effects, including wound healing and innate antimicrobial responses [173–176]. In fact, IL-22 activates STAT3-dependent transcription in human KC and upregulates the expression of proinflammatory and antimicrobial molecules such as S100 proteins, which are associated with the alternative differentiation pathway of KC, chemotaxis of T cells, neutrophils and monocytes, and even inflammatory functions [177–180]. Interestingly, Th2-derived IL-4 and IL-13 inhibit the TNF- and IFN*γ*-mediated production of *β*-defensins by KC. Together with a disturbed skin barrier function, this antagonism explains the high susceptibility to bacterial and viral pathogens in AD lesions, especially to* Staphylococcus aureus* infections [181–183]. The abundant release of S100 proteins as potent AMP is not counteracting the susceptibility to skin infections [184, 185]. In fact, S100 proteins are suggested to have antimicrobial activities, particularly against* Escherichia coli* and other Gram-negative bacteria, whereas* S. aureus* appears presumably resistant against upregulated S100 levels [186, 187]. Moreover, macrophages and infiltrating monocytes normally directly contribute to the elimination of* S. aureus* via TLR2 engagement, but there is evidence that a general impairment of TLR2 expression or TLR2-mediated cytokine release exists in AD patients [188, 189]. Therefore, the question remains whether CD1c^+^ DDC might overcome the lack of microbial clearance in AD. Indeed, the broad expression of TLR makes DDC highly susceptible to bacterial antigens, and the enhanced IFN-*γ* release, especially in chronic AD lesions, could further amplify the immunologic activity in the context of combating infiltrating bacteria. In fact, DC derived from blood circulating monocytes of AD patients reveal no impairment of TLR2 function [190]. However, TLR engagement of DC in mouse AD skin orchestrates the induction of IL-10^high^, IFN-*γ* ^low^-producing regulatory Tr1 (type 1) cells [191]. Finally, the impact of microbial TLR2 ligands including* S. aureus*-derived proteins on the function of human DC in the context of AD currently remains unresolved [168].

## 4. Conclusion

It is clear that an altered DC function by the local inflammatory setting contributes to skin inflammation and pathogenesis. Although recent work provides insight into the immunoregulatory role of distinct DC subsets in inflammatory environments, further studies will be required to understand the complex molecular mechanisms balancing innate and adaptive immune responses in inflamed human skin. This will eventually pave the way for novel anti-inflammatory therapies in skin diseases.
